# Undergraduate deficits in plastic surgery exposure and awareness of the specialty: a systematic review

**DOI:** 10.1308/rcsann.2023.0099

**Published:** 2024-02-16

**Authors:** H Bhachoo, SC Glossop, LR Mattey, C Pearson, L Hoade, N Cereceda-Monteoliva, L Scourfield, AT Poacher

**Affiliations:** ^1^ Wye Valley NHS Trust, UK; ^2^ Cardiff University, UK; ^3^ Cardiff and Vale University Health Board, UK; ^4^ Guy’s and St Thomas’ NHS Foundation Trust, UK; ^5^ King’s College Hospital NHS Foundation Trust, UK

**Keywords:** Education, Medicine, Plastic surgery

## Abstract

**Introduction:**

Plastic surgery is an important specialty that involves widespread medical knowledge, some of which is taught in undergraduate curricula. The General Medical Council provides a well-defined plastic surgery curriculum for postgraduate training. However, there is no consensus on the provision for undergraduates in this specialty, potentially giving rise to a deficit in undergraduate medical education and a suboptimal basis for plastic surgery postgraduate training. Our aim was to identify the gap in undergraduate plastic surgery teaching and to understand student perceptions of the specialty as well as any trialled interventions.

**Methods:**

A prospectively registered systematic review was conducted following the PRISMA (Preferred Reporting Items for Systematic Reviews and Meta-Analyses) guidelines. The MEDLINE^®^, Embase™, PubMed^®^ and Google Scholar™ databases were searched for literature relating to undergraduate exposure to plastic surgery and relevant teaching interventions. Ten studies were included in this review, categorised into three main themes: exposure during medical school, determining factors and perceptions for pursuing a plastic surgery career, and teaching interventions.

**Results:**

Surveys assessing medical student perceptions indicate a significant deficit in exposure to plastic surgery in the undergraduate curriculum. Medical students’ interest in the specialty is affected by multiple factors, including the amount of surgical exposure in medical school. Interventions to address the deficit mostly involve one-day courses.

**Conclusions:**

Although the literature is currently limited, studies are needed to effectively assess the outcomes of plastic surgery teaching methods in undergraduate training. Moreover, there is a need for consensus around the provision of undergraduate teaching in plastic surgery. This should be reflected in the latest undergraduate curricula in medical education.

## Introduction

Plastic surgery is a diverse and high-volume specialty that deals with the management of conditions affecting the body from head to toe, including burns, trauma, skin cancer, congenital conditions, hand surgery, breast surgery, and reconstruction of soft tissue defects from pressure sores, leg ulcers and severe infections.^1^ There is a significant crossover between the generalist and other surgical specialties, and so a basic understanding of plastic surgery should be provided to all undergraduates to ensure a base of knowledge in the management of these conditions. A vast body of knowledge and technical skills specific to the specialty is required of trainees in order to become a plastic surgeon. These range from principles of wound healing and scar management to challenging surgical techniques of skin grafting, tissue expansion, flap surgery and microsurgery.

Developing proficiency in plastic surgery requires not only a sound understanding of ubiquitous preclinical concepts such as physiology, immunology and anatomy but also the application of fundamental plastic surgery principles such as preserving vascularity, replacing tissue like for like, respecting anatomic zones and protecting tissue healing by careful surgical technique. However, although sufficient understanding of these topic areas is vital for the plastic surgeon, a general understanding of conditions relating to plastic surgery is also important for safe and effective general and specialty care by surgeons, medics, generalists and emergency practitioners.

There is a high level of competition for a career in plastic surgery,^2^ which highlights its popularity and the importance of a high-quality training programme to produce the very best surgeons. While postgraduate training is well defined,^3^ early exposure to a specialty is a key predictor of likelihood of choosing that specialty. Furthermore, many specialties will have to deal with conditions relating to plastic surgery throughout their careers^4^ and it is vital to have a foundational understanding of the core themes of the specialty to enable patient safety.

Although specific guidelines can be found in the Royal College of Surgeons England 2015 syllabus,^1^ there remains a lack of clarity and direction as to the optimal provision of undergraduate plastic surgery teaching. In addition, variance in teaching methods across medical institutions has resulted in significant deficits in the provision of basic surgical teaching for medical students,^5–7^ and any deficit in undergraduate training could potentially have an impact on preparedness and required basic knowledge for clinical practice after qualifying with an undergraduate medical degree.^8^

Consequently, it was our aim to perform a systematic review of the existing studies on undergraduate plastic surgery teaching in the UK. This comprises the amount of time that medical schools delegate to the teaching of plastic surgery topics, and the quality of such teaching as perceived by medical students and through assessment performance. We also sought to appraise the literature that discusses any alternative approaches that may have been trialled or used to teach medical students. This will enable an informed discussion relating to any potential reported deficits as well as alternative methods that may be useful to improve and strengthen curricula in medical schools across the UK.

## Methods

This review was prospectively registered on the PROSPERO database (CRD42023433881). The PRISMA (Preferred Reporting Items for Systematic reviews and Meta-Analyses) guidelines were followed throughout this review.^9^

The MEDLINE^®^, Embase™, PubMed^®^ and Google Scholar™ databases were searched for suitable literature. The search terms were (“medical student” OR “student” OR “undergraduate”) AND (“plastic surgery”) AND (“prepared” OR “confidence” OR “knowledge” OR “approach” OR “teaching method”) AND (“UK” OR “United Kingdom”). Limits included original research articles written in English within the past 20 years.

This review did not require ethical approval as it involved looking at previously published literature.

### Inclusion and exclusion criteria

Articles involving medical students at any point in their medical programme in the UK were considered. The focus of this review was understanding the provision of undergraduate education involving plastic surgery teaching. Consequently, studies that analysed student satisfaction, knowledge, skills and attitudes regarding plastic surgery were included. However, in order to be included in the evaluation and comparison of teaching methods, a study had to describe or evaluate an undergraduate teaching intervention where developing knowledge, skills or competences in plastic surgery were the primary outcome. Full details of the inclusion and exclusion criteria are detailed in Table 1.

**Table 1:** Inclusion and exclusion criteria for article eligibility

Criteria	Inclusion	Exclusion
Research participant	Studies evaluating an undergraduate plastic surgery learning curriculum, either knowledge or skill-based	Research relating to a research participant other than plastic surgery teaching
Participants	Studies based in the UK	Any studies from non-UK medical schools
Location	UK only	Any studies from outside the UK
Type of studies	Original and primary research	Commentaries, abstracts, conference abstracts, letters to the editor
Methodology	Quantitative/qualitative/mixed methodology	
Timescale	Literature published between 2003 and 2023 (past 20 years)	Literature published before 2003

### Title and abstract review

Following the exclusion of duplicate articles, two authors (HB and LH) independently reviewed titles and abstracts. Articles for full-text review were placed in a shared Excel^®^ spreadsheet (Microsoft, Redmond, WA, US) if the inclusion criteria were satisfied. For any discrepancies that arose in this process, a discussion was held to establish a consensus among both authors. All abstracts without consensus on initial eligibility were independently reviewed by two other authors (LM and CP) to determine whether they met the inclusion criteria.

### Full-text review and data extraction

A data extraction tool was created using the BEME (Best Evidence Medical Education) guidelines.^10^ Using this tool, two reviewers (LM and CP) independently read the full texts of the selected studies. Where discrepancies arose, an additional reviewer panel (HB and SG) read the full-text article and a consensus was reached, with any further queries brought to the senior author (AP). Extracted data were placed in the aforementioned spreadsheet.

Fields in the spreadsheet included description of teaching intervention, skills taught, educational setting, methods of data collection, and significance/implications and limitations. Educational settings were classified as clinical, classroom, simulation, online, project-based or mixed. Curricula taught in clinical settings took place in patient care environments. Curricula with mixed settings were those that used multiple settings, such as didactic education delivered in a classroom combined with project-based learning occurring outside the classroom.

**Figure 1:**
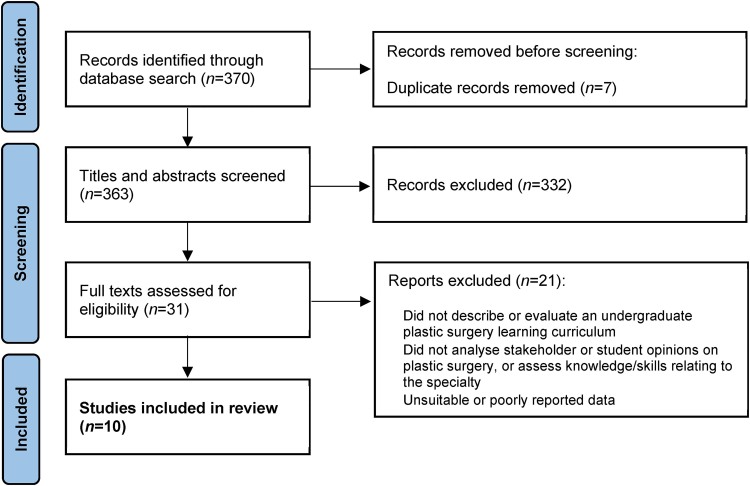
Flowchart of study selection

On completion of data extraction, ten articles met the inclusion criteria (Figure 1). These articles were scored using the medical education research quality instrument (MERSQI)^11^ to enable an informed evaluation of their research methods and the quality of the studies, which allowed appraisal of bias in a narrative synthesis. This scoring system has six domains (study design, sampling, type of data, validity of evaluation instrument, data analysis, outcomes) with five domains having a minimum score of 1 and all domains having a maximum score of 3. This gives a possible score range of 5–18, with 18 indicating the highest research quality.

## Results

Ten papers met the inclusion criteria and were incorporated in this review.^12–21^ There were three main themes: exposure during medical school, determining factors and perceptions for pursuing a plastic surgery career, and teaching interventions.

The mean MERSQI scores for all ten papers was 10.5 (standard deviation: 1.02), with a minimum score of 9 and a maximum score of 12.5 (Table 2). The mean of 10.5 indicates moderate research quality across the literature included in this review.

**Table 2:** Medical education research quality instrument (MERSQI) scores^11^ for the ten papers included in the review

Paper	MERSQI score
Zinchenko, 2016^12^	10.5
Farid, 2017^13^	11
Higgins, 2020^14^	10
Davis, 2016^15^	10.5
Kidd, 2021^16^	10
Dean, 2016^17^	9
Khatib, 2015^18^	11.5
Davis, 2010^19^	12.5
Leung, 2016^20^	11
Egro, 2017^21^	9

### Exposure during medical school

Our review suggests that the existing literature evaluating the current state of plastic surgery teaching is sparse. From the research that is available, it appears that there are concerning deficits in the amount of teaching in the medical curriculum. Zinchenko *et al* conducted a survey on the provision of burns teaching (a key area in plastic surgery) that received 348 responses from final-year medical students across the UK.^12^ Only 36% of respondents stated that burn management was part of their medical school curriculum and 70% of students had received no formal teaching by the time they had entered their final year. Among those who received teaching, the most popular method of teaching was a didactic lecture or seminar (51%). Poor coverage of plastic surgery in the UK medical curriculum may correlate with fewer students being interested in pursuing a career in the field.

A study by Farid *et al* at Birmingham Medical School (where plastic surgery teaching is offered as “optional” self-directed learning modules with no patient contact) found that only 18% of respondents (30/171) received their plastic surgery knowledge through formal teaching.^13^ An overwhelming number of students instead acquired knowledge from non-moderated media sources.

Higgins and Thomson implemented a new plastic surgery curriculum at the University of Glasgow.^14^ Prior to this, the university had no formal plastic surgery undergraduate teaching. They also sought to understand students’ current knowledge of and attitudes towards the field. The most commonly recognised subspecialty was burns but this was only identified by 47% of students (75/160); this was followed by breast surgery (43%, 69/160). More reassuringly, 85 students (53%) wanted more exposure to plastic and reconstructive surgery, which subsequently increased to 98 students (61%) after a session introducing plastic surgery as a career, suggesting that the demand for plastic surgery teaching does indeed exist among students.

### Determining factors for pursuing a plastic surgery career

Davis *et al* provided questionnaires to medical students at two undergraduate national courses to assess data relating to plastic surgery.^15^ Using responses from 175 students, they found a strong link between hours of exposure to the field and interest in a plastic surgery career (linear coefficient = 0.12, 95% confidence interval [CI]: 0.08–0.17, *p*<0.0001; r^2^=0.15), with those who had >75 hours’ experience having an interest in plastic surgery that was significantly higher than those with ≤75 hours’ experience (89.2% vs 58.1%; mean difference: 31.1pp, 95% CI: 19.3–42.9pp, *p*<0.0001).

This is reinforced by the results of Kidd *et al*, who administered questionnaires to Scottish medical undergraduates.^16^ Only 66 (34%) of the 193 respondents had positive opinions of plastic surgery, with a greater number (*n*=105, 54%) expressing negative views towards the specialty. Two-thirds (70%) of students indicated that placements in plastic surgery were a factor determining their perceptions of the specialty.

Influential factors for pursuing a plastic surgery career have also been studied in graduate-entry medical students. Dean *et al* conducted a cross-sectional survey at Swansea University Medical School (4-year course), with 153 students responding to a combination of open and Likert-type questions.^17^ They noted that 41% of students in year 1 and 43% in year 3 were of the opinion that surgical exposure affected career choice, with 19% of all students believing that competition ratios for plastic surgery were very competitive. Despite a large majority in each year group (80%, 84%, 74% and 91%) feeling that plastic surgery is portrayed negatively in the media, 88% overall stated that this would not influence career choice.

Farid *et al* found that factors that discouraged students from pursuing a career in plastic surgery included a lack of subject interest, long training and long working hours (64%, 60% and 52% of students respectively).^13^

### Teaching interventions

Interest in and career aspirations for plastic surgery may be affected by certain teaching interventions that address deficits experienced in the usual medical curriculum. Table 3 summarises the four papers that describe the implementation of new plastic surgery teaching methods.^18–21^

**Table 3:** Summary of studies that implemented new plastic surgery teaching methods

Study	Design	Sample size	Data collection method	Intervention	Findings
Khatib, 2015^18^	Survey	39 students	Pre and post-course questionnaire	A one-day plastic surgery course involving talks and surgical skills sessions	13% increase in level of interest in plastic surgery after the course (*p*<0.005) Self-reported plastic surgery skills and knowledge: mean score increased from 2.67 to 4.04 after the course (50% increase)
Davis, 2010^19^	Survey	93 students	Pre and post-course questionnaire	A one-day plastic surgery course for medical students with multiple aspects including lectures, skills teaching, workshops and quizzes	Four key themes assessed, all seeing significant improvements after the course: 1. Plastic surgery knowledge (*p*<0.0001) 2. Awareness of the work of a plastic surgeon (*p*<0.0001) 3. Ability to perform basic plastic surgery skills (*p*<0.0001) 4. Career interest in plastic surgery (*p*<0.0001) Ability to identify plastic surgery operations: improvement of 37% after the course (*p*<0.01)
Leung, 2016^20^	Survey	35 delegates (majority were medical students but also some junior doctors)	Self-assessed confidence scores before and after intervention	A one-day plastic surgery course for medical students and junior doctors providing short lectures and surgical skill teaching	Confidence scores for instrument handling, suturing, local anaesthesia and skin lesion excision all increased significantly after the course (all *p*<0.0001), with an average improvement of 51% Self-assessed knowledge domains (wound assessment/management, suture selection, concepts of flaps) all increased significantly after the course (*p*<0.0001)
Egro, 2017^21^	Survey	18 students	12-question feedback survey relating to level of experience, interest in surgery and satisfaction with the tool	An e-learning tool designed to educate on the management of burns	2/18 respondents had previously experienced burns teaching 50% had an interest in pursuing a surgical career72% expressed interest in the introduction of an e-learning platform for basic burns management in the curriculumSatisfaction domains: ease of use (87%), usefulness (88%), relevance to curriculum (90%), clarity of content (78%), quality of content (83%), design (79%)

Khatib *et al* ran a one-day plastic surgery course for undergraduates that involved a series of talks and plastic surgery skills workshops.^18^ Participants were asked to score all surgical specialties on a scale of 1–10 based on their interest in the particular field (with 10 showing the highest interest), and while the number one career aspiration for the 39 delegates in both the pre and post-course questionnaires was plastic surgery, the post-course results demonstrated an increase of 13% in the level of career interest in this specialty (*p*<0.005). As part of a 5-point Likert scale, Khatib *et al* also assessed students’ self-reported plastic surgery ability and knowledge; the mean score increased from 2.67 before the course to 4.04 after the course (50% increase).

Another one-day plastic surgery course discussed by Davis *et al* produced several interesting findings.^19^ Questionnaire results from 93 medical students indicated that the most common sources of previous experience before the course were lectures (44% of students), closely followed by theatre experience (42%). Four key themes were assessed both before and after the course: plastic surgery knowledge, awareness of the work of a plastic surgeon, basic plastic surgery skills and career interest). All four themes showed statistically significant increases after the course (*p*<0.01) with a 37% improvement (*p*<0.01) in students being able to recognise operations performed by a plastic surgeon.

Leung *et al* demonstrated similar findings after a one-day plastic surgery course for medical students and junior doctors, the majority (73%) of whom were MBBS students.^20^ All self-assessed confidence scores for knowledge of wound assessment/treatment, suture selection and concepts of flaps showed statistically significant improvements after the course compared with before the course (*p*<0.0001). Additionally, all self-reported confidence scores for practical skills (instrument handling, suturing, local anaesthesia, skin lesion excision) increased significantly after the course (*p*<0.0001).

Addressing specific deficits in curricula may also be useful to improve outcomes for medical students’ knowledge and understanding of plastic surgery concepts. Egro developed an e-learning tool for basic burns management and 18 medical students answered a 12-question survey following completion of the online course.^21^ Multiple domains were surveyed, with all domains receiving either “good” or “very good” ratings. Usefulness of the course was rated at 88% and relevance to the medical curriculum was rated at 90%, with overall course satisfaction at 87%.

## Discussion

This review highlights poor exposure to plastic surgery in undergraduate medical education, with sporadic and varying curricula across the country that do not meet student expectations or national guidance. Although plastic surgery is still considered a highly competitive specialty,^22^ this lack of early potential could result in the specialty losing out on a cohort of gifted trainees who have not had sufficient exposure to the specialty to make their decision.^6,23^ Furthermore and most importantly, it demonstrates a significant lack of knowledge and understanding of the specialty by the generalist, which will inevitably lead to worse patient care and poorer outcomes when not managed in a specialist tertiary centre.

Many topics taught in undergraduate curricula such as vascular physiology, clotting, inflammation, cancer and anatomy are very relevant to the specialty. Despite this, these are rarely given contextual consideration in terms of how these can be applied in both a clinical and ethical context to plastic surgery.^24,25^

There has been limited research in the UK assessing the provision of plastic surgery and related outcomes. Our findings highlight some important points of discussion, one of these being the amount of exposure that medical students experience as part of the MBBCh/MBBS course.^18–21^ Plastic surgery teaching appears to be one of the most poorly taught specialties in the undergraduate medical curriculum, with student surveys indicating inadequate coverage of all areas of the specialty (both practical and theoretical) despite its multidisciplinary involvement.

Concerningly, even areas directly relevant to general practitioners, emergency physicians and dermatologists such as early identification and management of burns,^26^ devascularised limbs, amputations and flexor sheath infections seem to be lacking. These topics should form part of every medical practitioner’s base theoretical knowledge. In undergraduate curricula, plastic surgery teaching is often provided as an optional module despite increasing interest from students in having teaching provided. This could exacerbate the issue as these optional modules are likely to be taken by those who are already interested. However, pending changes to qualifying assessments in medical schools involving the newly formed Medical Licensing Assessment may help to rectify this issue by requiring a ubiquitous understanding of various specialties including plastic surgery.^27^

Student-based surveys have also provided insight into the perceptions of the specialty and factors that determine career interest, largely involving the amount of experience that undergraduates receive. There appears to be a strong correlation between hours of experience in surgery and an interest in pursuing it as a career.^8^ This also applies in the context of whether students are exposed to the specialty during clinical placements. Furthermore, while media coverage of plastic surgery is something that can influence patient decisions to undergo such procedures,^25^ medical student perceptions of the specialty seem to be relatively unaffected despite acknowledgement of its negative media portrayal.^17^

In terms of other contributing factors, burnout among surgical trainees is a significant issue in the UK,^22,28,29^ with long hours and training, and fear of such an issue appears to have a negative impact on students’ desire to pursue a career in plastic surgery. This is an interesting point that could potentially be addressed by educating students on future career pathways at an early stage.

One-day courses comprising relevant talks and skill workshops seem to be the most popular intervention to address deficits in plastic surgery education.^18–20^ These one-off events described in the literature all showed significant increases in confidence levels or course satisfaction through survey-based analyses. This provides a relatively straightforward platform for the improvement and development of an undergraduate plastic surgery curriculum as the introduction of a one-day event has the potential to inspire students as well as educate them on the specialty. Conversely, optional courses such as those described above are likely to be attended by delegates already interested in plastic surgery and none of the studies we looked at utilised methods that assessed long-term follow-up to objectively assess their efficacy.

It is also important to note that evaluating self-reported confidence levels, and providing surveys to attendees before and after the intervention can lead to response bias and the observer-expectancy effect. Better quality research is therefore warranted to provide further insight into the optimal surgical teaching methodology for plastic surgery in the undergraduate curriculum.

## Conclusions

While competition for plastic surgery training remains high, it is essential that teaching is of a high standard, beginning at undergraduate level. This will allow the UK to produce knowledgeable and confident medical graduates who will excel in whichever field they wish to pursue.

For plastic surgery, an increased focus on further undergraduate teaching could comprise one-day courses, e-learning modules and practical workshops as trialled in the literature. Such interventions would also serve to dispel the negative perceptions held by students as reported in the literature, which would otherwise turn away bright individuals from the field. In order to test the rigour of such interventions, future studies should look at ways to definitively measure the effectiveness of their teaching interventions. This would mean a focus not just on student satisfaction and confidence but also on testing student knowledge over short and long-term timeframes. This could take the form of written, multiple-choice or clinical examinations.
